# Heart Rate Recovery: Up to Date in Heart Failure—A Literature Review

**DOI:** 10.3390/jcm13113328

**Published:** 2024-06-05

**Authors:** Andreea Cozgarea, Dragoș Cozma, Minodora Teodoru, Alexandra-Iulia Lazăr-Höcher, Liviu Cirin, Adelina-Andreea Faur-Grigori, Mihai-Andrei Lazăr, Simina Crișan, Dan Gaiță, Constantin-Tudor Luca, Cristina Văcărescu

**Affiliations:** 1 Institute of Cardiovascular Diseases Timisoara, 300310 Timisoara, Romania; andreea.cozgarea@umft.ro (A.C.); alexandra.hocher@umft.ro (A.-I.L.-H.); andreeaadelinafaur@yahoo.com (A.-A.F.-G.); mihai88us@yahoo.com (M.-A.L.); simina.crisan@umft.ro (S.C.); dan.gaita@umft.ro (D.G.); constantin.luca@umft.ro (C.-T.L.); cristina.vacarescu@umft.ro (C.V.); 2 Department of Cardiology, “Victor Babeș” University of Medicine and Pharmacy, 300041 Timisoara, Romania; liviu.cirin@umft.ro; 3 County Clinical Emergency Hospital of Sibiu, 550245 Sibiu, Romania; 4 Research Center of the Institute of Cardiovascular Diseases Timisoara, 300310 Timisoara, Romania; 5 Medical Clinical Department, Faculty of Medicine, “Lucian Blaga” University, 550024 Sibiu, Romania

**Keywords:** heart failure, heart rate recovery, cardiac exercise stress test, autonomic nervous system

## Abstract

The rising prevalence of cardiovascular disease underscores the growing significance of heart failure (HF). Pathophysiological insights into HF highlight the dysregulation of the autonomic nervous system (ANS), characterized by sympathetic overactivity and diminished vagal tone, impacting cardiovascular function. Heart rate recovery (HRR), a metric measuring the heart’s ability to return to its baseline rate post-exertion, plays a crucial role in assessing cardiovascular health. Widely applied across various cardiovascular conditions including HF, coronary artery disease (CAD), and arterial hypertension (HTN), HRR quantifies the difference between peak and recovery heart rates. Given its association with elevated sympathetic tone and exercise, HRR provides valuable insights into the perspective of HF, beyond effort tolerance, reaching toward prognostic and mortality indicators. Incorporating HRR into cardiovascular evaluations enhances our understanding of autonomic regulation in HF, offering potential implications for prognostication and patient management. This review addresses the significance of HRR in HF assessment, analyzing recently conducted studies, and providing a foundation for further research and clinical application.

## 1. Introduction

Heart rate recovery (HRR) is a parameter that addresses the decrease in the heart rate following a physical exercise, reaching its resting heart rate values. It is typically measured as the difference between the maximum heart rate during effort and the heart rate at a given recovery time [1,2]. It serves as a precious tool for evaluating autonomic nervous system (ANS) imbalance and has been widely used in screening and quantifying cardiovascular risk and all-cause mortality in patients suffering from heart disease [3,4]. This process reflects the dynamic equilibrium and synchronized interaction between the reactivation of the parasympathetic nervous system and the withdrawal of the sympathetic nervous system [5].

Cardiac autonomic dysfunction is frequently linked to cardiovascular disease (CVD) and has been observed in individuals possessing such risk factors [6]. In these populations, autonomic dysfunction is predominantly characterized by diminished parasympathetic drive and increased sympathetic activity. This imbalance amplifies cardiac load, increases ventricular instability, and consequently augments the susceptibility for cardiac arrest, infarction, and sudden death. Therefore, the existence of autonomic dysfunction suggests a more deprived prognosis for individuals with cardiovascular disease and more specifically, with heart failure (HF) [7,8].

Although the recently updated guidelines from the European Society of Cardiology (ESC) explicitly classify acute and chronic HF in the realm of the left ventricular ejection fraction (LVEF), the approach fails to adequately address the diverse pathophysiological mechanisms, etiological factors, and associated comorbidities inherent to HF. Notably, patients diagnosed with HF exhibit substantial clinical similarities irrespective of their LVEF status, underscoring the inadequacy of relying solely on LVEF for complete classification and management strategies [9,10].

In conditions of impaired ventricular contraction, the sympathetic nervous system activation aims to enhance the cardiac output by accelerating the heart rate. Norepinephrine release via adrenergic pathways augments myocardial contractility and triggers activation of the renin–angiotensin–aldosterone system (RAAS), essential for maintaining adequate blood pressure and cardiac output. Chronic elevation in wall stress instigates myocardial hypertrophy and remodeling, alongside vasoconstriction, thus elevating afterload and boosting contraction while impairing myocardial relaxation. This feedback loop intensifies neurohormonal stimulation, exacerbating cardiac output compromise and perpetuating heart failure progression [11,12,13].

On the other hand, increased vagal tone can help protect against ischemia and arrhythmias by reducing heart rate and blood pressure. Modern therapies aim to regulate cardiac dysautonomia by increasing parasympathetic activity through interventions such as vagal nerve stimulation [14].

The purpose of this review is to provide an in-depth analysis of the existing data on HRR in heart failure patients, by identifying underlying correlations ranging from prognosis and mortality to clinical outcomes and echocardiographic evaluation. Additionally, it aims to elucidate the potential of the parameter as a diagnostic metric in detecting and/or characterizing HF patients.

## 2. Autonomic Influence in Exercise and the Recovery Period

Heart rate during physical exercise is influenced by both central (central command) and peripheral (metabo- and mechanoreflex) mechanisms [15]. The metaboreflex, a physiological reflex triggered during or after exercise-induced muscle ischemia, is activated by stimulation of metabo-receptors. In healthy individuals, this reflex mechanism enhances hemodynamic response, leading to increased cardiac output primarily through flow-mediated mechanisms [16]. Physical exercise causes intricate shifts between the sympathetic and parasympathetic branches of the autonomic nervous system, concluding in an augmentation of heart rate (HR) through vagal withdrawal and adrenergic discharge. This complex physiological response enhances cardiovascular parameters, including cardiac output, HR, cardiac contractility, alveolar ventilation, and venous return to the heart. As exercise intensifies, the peak of sympathetic discharge and catecholamine release induces vasoconstriction in various circulatory systems, with exceptions in muscle, coronary, and cerebral circulations [17]. Despite increased sympathetic activity during exercise, parasympathetic modulation continues to influence HR, ensuring optimal myocardial perfusion during diastolic intervals and contributing to cardioprotective effects [18].

The recovery phase after maximal exercise involves an obvious shift in autonomic tone, typified by sympathetic withdrawal and parasympathetic reactivation. Notably, parasympathetic reactivation promptly manifests within the initial minute following exercise cessation, significantly influencing HR decline. While vagal reactivation predominantly mediates the initial 30 s HR decline, the prominence of sympathetic withdrawal becomes more apparent two minutes post-exercise [6,19]. The available literature data highlight the hemodynamic changes that occur in the recovery phase, when the important HR drop may be caused by a decreased cardiac output, mediated by intrinsic regulation [20]. Autonomic influence marks both HRR and the metaboreflex. Available data reveal that HF patients exhibit an increased metaboreflex, caused by an increased sympathetic activity. This results in excessive arteriolar vasoconstriction and increased systemic vascular resistance. Consequently, ventricular contraction is compromised, leading to a reduced stroke volume. Similar changes were noted in diabetic patients or those with metabolic syndrome [21].

As opposed to heart failure, trained individuals maintain a higher cardiac output during the recovery phase, caused by the redistribution of blood to the central regions, leading to an increased preload [20]. Thus, along with the more augmented vagal activity, it explains the shifts in hemodynamics in a trained individual compared to heart failure states. While chronotropic incompetence (CI) itself serves as a marker of cardiac dysautonomia, conflictual data appeared regarding beta-blockers, that are commonly used to treat heart failure. To address this issue, a lower threshold has been introduced for patients taking beta-blockers, which defines CI as the inability to reach 62% of APMHR [22].

Exercise elicits a sophisticated autonomic response, involving sympathetic dominance and enduring parasympathetic modulation, thereby influencing the dynamics of heart rate and recovery. Considering that heart failure is a state of hypersympathetic activity, exploring the recovery phase contributes to a better understanding of the ongoing processes, allowing the evaluation of therapeutic approaches.

## 3. Main HRR Parameters

As early as the 1990s, the first data appeared concerning HRR, stating that a HRR < 12 beats per minute in the first minute strongly predicts overall mortality, setting a cornerstone for future studies [23]. Since then, the topic of HRR has been gaining interest among researchers, who developed various means of determining HRR, at different time intervals of the recovery period.

The following paragraph summarizes various HRRs, grouped through measurement methods.

### 3.1. Difference between Peak HR and HR at a Certain Moment of Recovery

- **HRR1**—HRR at 1 minute of recovery subtracted from the peak HR [24,25,26,27,28,29,30].
- **HRR2**—HRR at 2 min of recovery subtracted from the peak HR [25,28,29,31,32].
- **HRR150 sec**—measured as the difference between the maximum HR and the HR after 150 seconds of recovery [33].
- **HRR3**—measured as the difference between the maximum HR and the HR after three minutes of recovery [34].

### 3.2. A Ratio between Different Phases of the Effort

- **Heart rate recovery index**—measured as the ratio between heart rate acceleration time (AT) and heart rate deceleration time (DT) [35].

### 3.3. A Delay in the Maximum Heart Rate

- **Delay of peak HR**: HRR assessment 6 months after heart transplantation reflects the cardiac denervation and the loss of vagal tone, which would normally induce HR drop after exercise [32].

Given the distinct recovery phases and autonomic involvement, evaluating sympathetic nervous system activity during the post-exercise recovery phase could be crucial, given the correlation between cardiovascular issues induced by exercise and raised sympathetic activation. Moreover, while numerous population-based studies have associated HRR with mortality, there remains a gap in research regarding whether the augmentation of HRR following a therapeutic intervention serves as a predictive factor for improved survival during the subsequent follow-up period [36].

## 4. Clinical Applications of HRR

Ideas regarding HRR have been widely used in various clinical settings besides coronary artery disease. Heart failure, which will be thoroughly discussed further, has intricate connections with dysautonomia, implicit with the consequences of hypersympathetic states. Autonomic nervous system dysfunction is also implied in conditions such as hypertension [3], diabetes mellitus (DM) [37], metabolic syndrome and obesity [38], and obstructive sleep apnea syndrome [39].

HRR evaluation in DM plays a significant role in detecting subclinical LV dysfunction and preventing HF by evaluating diastolic functional reserve among heart failure with preserved ejection fraction (HFpEF) risk factors [40]. In a study by Vukomanovic et al., both type 2 diabetes patients and healthy individuals were enrolled to evaluate autonomic cardiac dysfunction using CPET. The results of the study revealed that patients with type 2 diabetes displayed a blunted HRR, which was associated with altered HRR, yielding significant autonomic nervous system dysfunction [41]. Moreover, another piece of research revealed the importance of dysautonomia by highlighting the associations between diastolic dysfunction and diminished functional capacity in patients with type 2 DM [42].

A recent study involving 2540 patients aimed to examine the correlations between heart rate recovery (HRR) and terminal-pro B type natriuretic peptide (NT-pro-BNP), which are both established as prognostic markers for heart failure. The study found that the NT-pro-BNP value was correlated with HRR2 and HRR3, indicating the importance of HRR in the slow recovery phase. This suggests that NT-pro-BNP is linked to the sympathetic nervous system, which is associated with heart failure processes. The study highlights the significance of cardiac dysautonomia evaluated by HRR and its connection to strong prognostic indicators of heart failure [43].

## 5. Methodology

The current review aims to encompass the most relevant studies conducted in the last ten years, concerning HRR in HF patients, employing two tables that hold the most relevant aspects of the studies (Table 1 and Table 2).

The methodology used for this review implied searching on different databases, such as PubMed or Google Scholar, using relevant keywords like “heart rate recovery” and “heart failure”, including Mesh terms.

The criteria for inclusion stipulated that the articles be written in English and published between 2014 and 2024, and their type should relate to patients involved in clinical studies. Furthermore, the resulting articles were manually selected considering the relevance, intending to showcase diverse clinical scenarios where HRR was employed to address heart failure.

## 6. HRR in Heart Failure: Bedside Studies

The resulting population heterogeneity prompted the construction of two tables to effectively organize the resulting data. Table 1 presents patients diagnosed with heart failure with reduced ejection fraction (HFrEF), offering a focused examination of this subgroup within the cohort.

In contrast, Table 2 encompasses a diverse range of study populations and comparison groups, including heart failure with preserved ejection fraction (HFpEF), heart failure with mid-range ejection fraction (HFmrEF), and comparisons between heart failure patients and healthy controls. Furthermore, two specific studies are highlighted within this framework: the study of Carneiro et al. [34], which investigated individuals at risk of developing heart failure (Stage A), and the study of Imamura et al. [32], which delves into patients recovering from advanced heart failure (Stage D), and having undergone heart transplantation.

HRR is a powerful tool for predicting morbi-mortality in various conditions and is considered a marker of cardiac dysautonomia [44].

Table 1 compresses studies conducted on HFrEF patients and reveals several HRR methods of determination, exercise protocols, and controversial aspects such as beta-blockade treatment, alongside the study objectives and results.

Our goal is to assess the various purposes for conducting exercise tests, which range from assessing the impact of reduced quality of life to establishing correlations with mortality in connection with blunted heart rate recovery. We also aim to point out the correlation between reduced heart rate recovery and pro-inflammatory states, as well as the association between exercise tests and conditions such as sarcopenia, CRT responsiveness, and the influence of cardiac rehabilitation.

Delving into HRR determination methods, the most frequently used methods are represented by HRR1 and HRR2. Cozlac et al. used another determinant, namely the heart rate recovery index (HRRI) [35]. This index evaluates both the exercise time, by assessing the heart rate acceleration time (AT), and the time of recovery, by measuring the heart rate deceleration time (DT), being defined as the ratio between AT and DT.

Although the study population is represented by HFrEF patients, and the HRR determinants are equal, cut-off values either differ between studies or have no values available for cut-off, showing a degree of inconsistency. Moreover, additional differences among the studies are evident regarding the exercise test employed, the protocols implemented, and the duration of the recovery period. These studies have employed diverse exercise protocols and test types, spanning from the six-minute walk test (6 MWT) to comprehensive cardio-pulmonary exercise tests (CPET). The recuperation period varies across studies, encompassing active recovery followed by passive recovery, solely passive recovery protocols, or instances where the recovery protocol is not specified.

Similar to a recently published study, the methodology did not exhibit a clear pattern for which exercise type and recovery protocols were used, indicating that further research and studies are necessary to validate distinct protocols for various clinical scenarios [2].

The exercise types varied from the 6 MWT to the treadmill and cycle ergometer. While maximal exercise tests like CPET are considered the benchmark for evaluating functional capacity, the 6 MWT can offer valuable insights into a patient’s daily activity levels and short-term prognosis, particularly in those with heart failure and reduced ejection fraction, whether in a stable chronic condition or following an acute decompensation [45].

In contrast to the 6 MWT, both the cycle ergometer and treadmill offer a more gradual and graded exercise. The bicycle ergometer has several advantages, including a small space requirement, easy quantification of exercise, and suitability for obese patients or those with orthopedic disorders. From a safety standpoint, it is a better choice than other forms of exercise equipment, as it reduces the risk of ventricular arrhythmias and angina pectoris appearance during exercise. The reason for this is that initially, maximal exercise on a treadmill exerts more significant stress on the heart and lungs compared to bicycle ergometers. Research has shown that during exercise tests with a bicycle ergometer, untrained individuals often end the test due to fatigue in the quadriceps femoris muscles, resulting in an average 5–20% lower peak oxygen consumption (peak VO2) compared to treadmill exercise [46].

**Table 2:** Heart rate recovery (HRR) studies including mixed groups of heart failure (HF) patients and healthy controls.

Study (Year)	Patients Enrolled (n)	HF Population	Purpose of the Study	Exercise Test Methodology	Beta-Blocker Treatment	HRR Evaluation Method and Cut-Off	Conclusions
Hossri et al. (2024) [24]	106	HFpEF and HFmrEF with concomitant CAD	Benefits of CPMR in HF patients with CAD	Treadmill CPET using an incremental maximal protocol, followed later by a submaximal constant load protocol at 80% of the initial test; recovery period of 6 min	87% of the patients. Dosage N/A.	HRR1 was evaluated at the first recovery min; Cut-off N/A	12 weeks of CPMR were associated with improved NYHA class, significant exercise test performance, an increased HRR, and an enhanced QOL.
Irfanullah et al. (2023) [47]	39	HFpEF, HFmrEF, HFrEF	The effects of cycle ergometer training on heart rate recovery and mindfulness in patients with NYHA Class I and II heart failure	6 MWT on a 400–700 m distance.	All patients received either beta-blockers or CCB. Dosage N/A.	HRR1; HRR2 = HRmax-HR at 1; 2 min of recovery; Cut-off N/A	HRR1 and HRR2 improved after 6 weeks of cycle-ergometer training, as well as the MAAS.
Carneiro et al. (2021) [34]	2066	Participants without HF	Incidence of HF and its type (HFpEF and HFrEF) during the follow-up period (16.8 years)	Submaximal treadmill exercise test using the Bruce protocol; recovery in a supine position.	N/A	HRR3 = HRmax-HR at 3 min of recovery; Cut-off N/A	Slower HRR3 is associated with a higher risk of developing heart failure, particularly HFrEF.
Hajdusek et al. (2017) [33]	103	78 advanced HFrEF and 25 healthy controls, assessed for device implantation or transplant eligibility	Evaluation of HRR and MCR as outcome determinants in HF, during a follow-up of ~3.4 years	Symptom limited using a bicycle CPET, with a 25 W increase/3 min.	97% of HF patients. Daily low-middle doses (12.5–50 mg Carvedilol; 2.5–200 mg Metoprolol and 2.5–10 mg Bisoprolol)	The difference between HRmax and HR at 150 s of recovery; Cut-off N/A	MCR slope correlates with distinct clinical variables compared to HRR. In heart failure patients, the MCR slope offers significant prognostic value beyond HRR.
Yaylalı et al. (2015) [31]	41	HFrEF and HFmrEF	Correlation between exercise training and HRR improvement, before and after entering a training program	Symptom limited bicycle CPET with 10 W/min followed by 3 min active cool-down	51.2% of patients. Dosage N/A	HRR1; HRR2 = HRmax-HR at 1; 2 min of recovery; N.V. Preestablished HRR1 > 12 bpm; HRR2 > 22 bpm	The training enhanced only HRR2, with IT showing a greater impact on the HRR2 improvement. Both HRR (1 and 2) in those with an abnormal HRR baseline have improved after exercise.
Imamura et al. (2015) [32]	21	Heart transplant patients	Impact of post-heart transplantation parasympathetic reinnervation	Symptom limited Cycle-ergometer CPET using a 10 W/min incremental protocol, followed by a 5 min passive recovery in a seated position.	19% of patients. Dosage N/A.	HRR2-the difference between peak HR and HR after 2 min of recovery (measured 2 years after transplant) Delay of peak HR-the delay from the peak HR after the recovery time initiation (measured 6 months after transplant). Cut-off N/A	Parasympathetic reinnervation coincides with enhanced post-exercise recovery and heart failure-specific quality of life during the 2 years following heart transplantation.
Lindemberg et al. (2014) [26]	161. 154 completed the test	126 patients with HFrEF and 35 healthy individuals.	Correlations between HRR1 and 6 MWD	6 MWT followed by passive (seated) recovery.	70.58% of HF patients. Carvedilol mean dose 30 ± 29 mg.	HRR1 = HRmax-HR at 1 min of recovery; N.V. Preestablished HRR1 > 12 bpm	HF patients who received beta-blockers had better exercise tolerance than those without receiving beta-blocker medication, even though they had altered HRR.
Cahalin et al. (2014) [30]	240	200 patients with HFrEF and 40 patients with HFpEF	Correlations between HRR measured post 6 MWT and CPET and predictors of abnormal HRR.	6 MWT followed by a passive recovery. A symptom-limited bicycle CPET lasting 8–10 min. 1 min active cool-down period.	60% of the entire study group. Dosage N/A	HRR1 = HRmax-HR at 1 min of recovery; N.V. Preestablished HRR1 > 12 bpm	The 6 MWT and CPET were correlated concerning HRR, HR Reserve, and peak HR. Predictors of abnormal HRR were found to be Peak HR, EOV and E/e’ ratio.

N—Number; HF—heart failure; HFpEF—heart failure with preserved ejection fraction; HFmrEF—heart failure with mid-range ejection fraction; HFrEF—heart failure with reduced ejection fraction; CAD—coronary artery disease; CPET—cardiopulmonary exercise test; CPMR—cardiopulmonary and metabolic rehabilitation; N/A—Not available; HRR—heart rate recovery at a given time; HRmax—maximum Heart Rate; Min—minute; N.V.—Normal Values; S—Second; B.P.M.—beats per minute; NYHA—New York Heart Association; QOL—quality of life; 6 MWT—6 min walk test; 6 MWD—6 min walk distance; CCB—calcium channel blockers; MAAS—mindful attention aware scale; W—watt; MCR—metabolic chronotropic relation; EOV—exercise oscillatory ventilation.

The study’s population distribution is shown for example in the studies of Youn et al. or Cozlac et al. studies. The former implied symptom-limited CPET that was conducted on patients who presented with acute decompensated heart failure (ADHF), after the clinical stabilization of the patients [29]. The latter showed that an increased HRRI was associated with responder status, while a blunted HRRI was linked to non-responders in CRT patients [35].

The presented data from the numerous studies have covered diverse study groups, comprising patients from all HF stages [48]. HRR was studied in numerous population groups, extending from HFrEF and HFmrEF to HFpEF, including cohorts of patients who were at risk of developing HF in the follow-up period, as evaluated by Carneiro et al. The study evaluated the patients enrolled in the Framingham Offspring Study to assess the association between decreased HRR and incidental HF during an average follow-up period of 16.8 years. Considering that nearly 40% of the patients were on antihypertensive treatment, 16% on lipid-lowering treatment, 12% were smokers and 9.3% were suffering from type 2 Diabetes Mellitus (T2DM), they could be considered at risk for developing HF (Stage A heart failure) [48]. Thus, it is noteworthy to mention the correlation between blunted HRR and an increased risk of developing HF, particularly HFrEF [29,34,35].

Conversely, Imamura et al. comprised the opposite spectrum of HF patients, namely those who underwent a heart transplantation, underlining the broad range of HRR applicability [32].

Research has suggested that cardiac rehabilitation programs may have a beneficial effect on HRR. Several studies have indicated that there is a significant improvement in HRR following enrolment in a rehabilitation program [24,27,34]. Not only did rehabilitation result in improved HRR, but CAD patients were shown to have attenuated exercise-induced arrhythmias and less frequent myocardial ischemia responses. Moreover, HRR appears to be correlated with other HF determinants, such as the NYHA class, the BORG scale on exertion perception, and quality of life questionaries [27].

It is common for patients with heart failure to experience mental health concerns like anxiety and depression, which can sometimes be underestimated. Research conducted by Irfanullah et al. has revealed that cardiac rehabilitation not only enhanced HRR but also has a considerable positive effect on mindfulness scale assessment. This is a crucial finding as mindfulness has been shown to promote an increase in vagal stimulation, which is advantageous for heart failure patients and may help to improve their mental and physical well-being [47].

Controversial aspects are contained in the tables, regarding the beta-blocker (BB) treatment and its influences on HRR determinations. β-blocker therapy leads to a decrease in neurohormone levels and an increase in β-receptor sensitivity, ultimately resulting in accentuated inotropy. Additionally, it has been suggested that, among other medications, β-blockers may experience reduced efficacy over time [49].

HRR was not statistically different in patients who were on BBs, compared to those who did not, as stated in several studies [2,29]. This suggests that HRR predominantly reflects vagal tone and remains a viable method for risk stratification across all patients, regardless of β-blocker therapy [2].

In the study of Hajdusek et al., a statistical difference was observed in the baseline characteristics of patients concerning beta-blockers. This was performed by comparing individuals with heart failure with reduced ejection fraction (HFrEF) who were administered beta-blockers (BBs), and healthy controls who did not have any medical reason for receiving BBs. However, HRR was found to reflect the vagal tone and was not associated with BB usage [33].

Yaylali et al. studied HF patients who were enrolled in a cardiac rehabilitation program, pointing out that significantly more patients on BBs had normal baseline HRR1 and HRR2, before starting the rehabilitation program [31].

Lindemberg et al. conducted a study with patients divided into three groups: G1 comprised HF patients treated with BB, G2 consisted of HF patients not treated with BB, and G3 included healthy patients. Even though the patients who received BB had an altered HRR, they showed better exercise tolerance, according to the study’s findings [26].

Various studies that determined HRR are displayed in the tables, but the methods they used were not consistent. Most studies rely on HRR1 evaluation using a cut-off value that was put in place over 30 years ago. However, two studies used statistical methods to evaluate their HRR cut-off; Cozlac et al. referred the HRRI value to the area under the receiver operating characteristic (AuROC) [35]. Likewise, Youn et al. stood out as they use Contal and O’Quigley’s method to establish a cut-off value [29]. In contrast, other studies do not have a clear cut-off value, or they depend on preexisting cut-off values.

**Figure 1:**
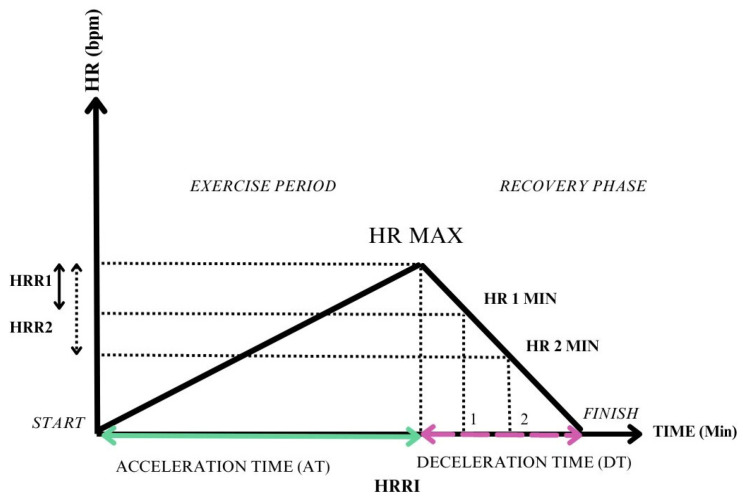
Graphical interpretation of heart rate recovery parameters. HRR1: HR MAX- HR1min (heart rate recovery at 1 min); HRR2: HR MAX-HR2 2 min (heart rate recovery at 2 min); HRRI: AT/DT (heart rate recovery index).

Figure 1 shows a graphical representation of the parameters used for assessing HRR. Whereas the most used evaluation methods use the difference between peak HR and HR at a certain moment of recovery (HRR1 and HRR2), a ratio between the exercise phase and the recovery period, such as HRRI, is also displayed. The significant benefit of using HRRI is that it takes into account the entire exercise period and reports a single value that assesses both acceleration and deceleration time.

## 7. Echocardiographic Correlations

Echocardiography is a significant part of cardiovascular assessment. The following section contains research data that revealed associations between certain echocardiographic determinants and HRR.

LVEF is a cornerstone parameter for evaluating systolic function, yet its exclusive use is inadequate, particularly given the increasing prevalence of HFpEF. Comprehensive assessments now require additional evaluations, including diastolic function assessment, tissue Doppler imaging (TDI), and evaluation of left atrial enlargement [10]. Researchers have revealed that an examination of cardiac hemodynamic parameters, particularly through tissue Doppler echocardiography, indicates a clear correlation between left ventricular diastolic function and HRR. Patients showing decreased left ventricular filling pressures and exhibiting lower E/e’ ratios demonstrate accelerated HRR and a more pronounced chronotropic response, underscoring the intricate relationship between cardiac autonomic function and adaptive responses to exercise [50]. As previously stated in the HF bedside studies, Cahalin et al. revealed that echocardiography can serve as a predictor for HRR values, involving mainly the E/e’ ratio for evaluating the diastolic filling pattern [30]. 

Whereas conventional echocardiography has a certain contribution, diastolic assessment during stress echocardiography has shown superior results to conventional echocardiography and has been widely evaluated and incorporated in HFpEF diagnostic scores. The most evaluated parameters represent peak tricuspid regurgitation velocity jet and subsequently, pulmonary artery systolic pressure (PASP) and mitral E/E’ to objectify elevated LV filling pressures [51].

The relationship between HRR and left atrial function and size is worth mentioning. Interestingly, blunted HRR has been shown to relate positively to the left atrial volume index, indicating that cardiac dysautonomia mechanisms may contribute to cardiac remodeling processes [51]. As shown in a recent study, abnormal strain rates during the reservoir, conduit, and contraction phases of the left atrium were associated with blunted HRR 120 s in patients with ST-segment elevation myocardial infarction [52]. 

## 8. Discussion

This current review discussed the topic of HRR and the main studies that involved HF patients, in which various aspects and correlations of HRR were determined. The main HRR studies that were conducted in the last decade underscore the inconsistency of HRR determinants, underlining that each method has its weaknesses and strengths.

Since the recovery phase is influenced differently by the autonomic nervous system, we consider that it is crucial to comprehend the whole-effort dynamics to better assess heart failure conditions.

Ranging from chronic inflammatory states, metabolic imbalance, and sarcopenia to cardiac outcomes, HF predictability, and mortality, we encompassed the various applicability of HRR in our presented study.

Available data suggest facilitated patient risk quantification with the help of HRR, considering that patients with blunted HRR indices exhibit increased risks of mortality, recurrent HF hospitalizations, and adverse cardiac events [25,29,44]. By identifying high-risk individuals, we presume that clinicians could adapt management strategies accordingly to HRR values.

Another key determinant of cardiac dysautonomia is reflected by heart rate variability (HRV). HRV refers to the variation in the time intervals between successive heartbeats (i.e., the R-R interval) and has been proven to have decreased values in HF [53]. While they both serve as prognostic markers [54,55], they differ in their measurement methods and the information they provide. Firstly, HRV is usually measured on standard resting electrocardiograms (ECG) or during Holter monitoring [56]. HRR on the other hand, is measured after a graded exercise and offers more complex information about both the resting HR and the recovery HR at a given time.

The mutual regulation system observed between HRV and HRR implies a potential connection between the two measures. If so, resting HRV indicators could serve as predictors of HRR before encountering stressors like exercise [57].

However, the diagnostic utility of HRR remains to be further proven, as it would be a crucial aspect to implement HRR among well-established tools, such as natriuretic peptides, in the diagnostic of heart failure.

Exercise stress tests have been helping clinicians evaluate treatment response in advanced HF states, that require CRT. A recently published article underlines the importance of ET in tailoring beta-blockers and ivabradine in CRT fusion pacing. Monitoring dynamic changes of the HR over time can help evaluate the efficacy of treatment regimens and guide adjustments as needed [58]. Therefore, our perspectives may shift towards better HRR comprehension, which may be useful in guiding medical treatment in HF patients. Therapeutic interventions aimed at improving cardiac function, such as medication adjustments, lifestyle improvements, and cardiac rehabilitation programs, may lead to enhancements in autonomic function and subsequently improved HRR.

As previously exposed, exercise training represents a cornerstone of HF management, promoting cardiovascular fitness and improving symptoms, further enhancing the quality of life of HF patients. Among improving cardiovascular fitness, exercise modulates dysautonomia, improves inflammatory states, and prevents muscle loss, which has been proven to be a consequence of the pathophysiology of heart failure [59].

Therapeutic interventions targeting autonomic modulation vary widely, starting with medical treatment, such as beta-blockers, angiotensin-converting enzyme inhibitors (ACEIs), or digoxin. More recently, studies have shown that sodium-glucose cotransporter-2 inhibitors (SGLT2-i) may improve cardiac autonomic dysfunction in type 2DM patients, as published in the SCAN Study, which may lead to new research directions toward HF patients [60]. Besides medical therapies, dysautonomia may be targeted with device-based options, such as CRT, vagal nerve stimulation (VNS), or renal denervation [61].

## 9. Further Perspectives

Assessing HRR in the treatment of HF patients will offer a simple approach to risk stratification, treatment monitoring, and proactive management.

HRR has been identified as a potential indicator of premature cardiac impairment, such as altered diastolic dysfunction, as demonstrated in a study involving obese adolescents. This underscores its significance as a clinically relevant outcome marker [62]. Although the available determination methods prove some degree of inconsistency with the used parameters, the field is open for novel implementations.

Future studies may show interest in establishing connections between abnormal HRR, HF, and atrial remodeling. Considering the intricate synergy between dysautonomia, electrical and mechanical remodeling, and heart failure progression, the area of research deserves to be unveiled.

Another key aspect concerns inflammation, frequently measured by high-sensitive C reactive protein (hs-CRP) [63] or the neutrophil-to-lymphocyte ratio (NLR) [64], which is known to be linked to the risk of developing cardiovascular disease, such as heart failure [65] and atrial fibrillation [66]. Further studies are necessary to evaluate the risk of quantifying atrial fibrillation in the realm of blunted HRR.

Moreover, delayed HRR has been proven to be independently associated with NLR, indicative of systemic inflammation [63]. This underscores the implications of vagal modulation in inflammation and suggests future perspectives for managing heart failure.

## 10. Conclusions

In summary, this study underscores the necessity of integrating heart rate recovery (HRR) into cardiovascular assessments, as it is associated with both morbidity and mortality in numerous cardiovascular conditions. The connections between HRR and the autonomic nervous system make HRR an accountable index, for both the assessment of the pathophysiologic dynamic processes and their consequences [67]. However, the domain of heart rate recovery stands as a focal point for future research endeavors, particularly in the context of heart failure, where the potential diagnostic utility of HRR remains to be fully elucidated. As mentioned before, the recently published studies encompassing HF patients lack a standardized evaluation protocol that could be easily applied throughout practices, emphasizing the importance of future research in this area of expertise.

Establishing the diagnostic power of HRR through further investigation holds significant promise, especially considering its ease of evaluation. Such advancements could revolutionize cardiovascular diagnostics, offering clinicians a simple yet potent tool for enhancing risk assessment and patient care.

## Figures and Tables

**Table 1:** Heart rate recovery (HRR) studies including patients with heart failure with reduced Ejection Fraction (HFrEF).

Study (Year)	Patients Enrolled (n)	HF Population	Purpose of the Study	Exercise Test Methodology	Beta-Blocker Treatment	HRR Evaluation Method and Cut-Off	Conclusions
Andrade et al. (2022) [25]	76	HFrEF	Correlation of all-cause mortality and HRR1 and HRR2 during a 2-year follow-up	6 MWT followed by passive supine recovery.	97% of patients. Dosage N/A.	HRR1; HRR2 = HRmax-HR at 1; 2 min of recovery; Preestablished N.V. HRR1 > 12 bpm; HRR2 > 22 bpm	Decreased HRR1 and HRR2 are associated with increased mortality.
Tanaka et al. (2021) [27]	84	HFrEF with AF	Correlation of HRR and exercise capacity of HFrEF and AF patients before and after the rehabilitation program	Cycle ergometer CPET using a ramp protocol of 10 W/min until exhaustion, with an active 1 min recovery, followed by a 4 min passive recovery.	90% of patients. Dosage N/A	HRR1 = HRmax-HR at 1 min of recovery. For AF patients, HR was determined by averaging the last ten beats at each point; Cut-off N/A	Improved HRR is associated with improved exercise capacity in patients with HFrEF and AF after completing the cardiac rehabilitation program.
Cozlac et al. (2020) [35]	109	HFrEF patients following CRT implantation	Correlation of HRRI and CRT responsiveness	Cycle ergometer using the Bruce Protocol with a 25 W increase/2 min.	82.8% of the patients. Dosage N/A.	HRRI = The ratio between HR AT and DT. The cut-off for CRT response predictability was 1.51.	HRRI was significantly higher in CRT responders vs. non-responders.
Fonseca et al. (2019) [28]	116	HFrEF	Association of sarcopenia and autonomic regulation	Symptom limited cycle ergometer CPET using a ramp protocol 5–10 W/min. Active recovery for 2 min, followed by 4 min of passive recovery.	100% of sarcopenic and 94% of non-sarcopenic patients. Dosage N/A.	HRR1; HRR2 = HRmax-HR at 1; 2 min of recovery; Cut-off N/A	Sarcopenia is associated with decreased HRR1 and HRR2 in HF patients.
Youn et al. (2016) [29]	107	Recovered acute decompensated HFrEF (Eligible for discharge)	Correlation between HRR and pro-inflammatory states with clinical outcomes	Treadmill CPET using a modified Bruce Protocol. Passive recovery in seated position.	Total of 33.3% in the CV-events group and 68.8% in the no-CV-events group. Dosage N/A.	HRR1; HRR2 = HRmax-HR at 1; 2 min of recovery; Cut off HRR1 < 13, HRR2 < 27	Impaired HRR is associated with an exaggerated pro-inflammatory response and independently predicts clinical outcomes.

N—Number; HF—heart failure; HFrEF—heart failure with reduced ejection fraction; AF—Atrial Fibrillation; 6 MWT—6 min walk test; HRR—heart rate recovery at a given time; HRmax—Maximum Heart Rate; HRRI—heart rate recovery index; CPET—Cardiopulmonary exercise test; Min—minute; S—Second; B.P.M.—Beats per minute; W—Watt; CRT—Cardiac Resynchronization Therapy; AT—Acceleration Time; DT—Deceleration Time; N.V.—Normal Values; N/A—Not available; CV—Cardiovascular.

## Data Availability

Data sharing is not applicable.
